# Robotic transhiatal revision of gastric conduit after esophagectomy: A technical overview

**DOI:** 10.1016/j.xjtc.2025.09.038

**Published:** 2025-10-30

**Authors:** SangMin Kim, James D. Luketich, Gabriella M. Lloyd, Marissa A. Matto, Ian G. Christie, Samuel L. Luketich, Evan T. Alicuben

**Affiliations:** Division of Thoracic and Foregut Surgery, Department of Cardiothoracic Surgery, University of Pittsburgh Medical Center, Pittsburgh, Pa

**Figure:**
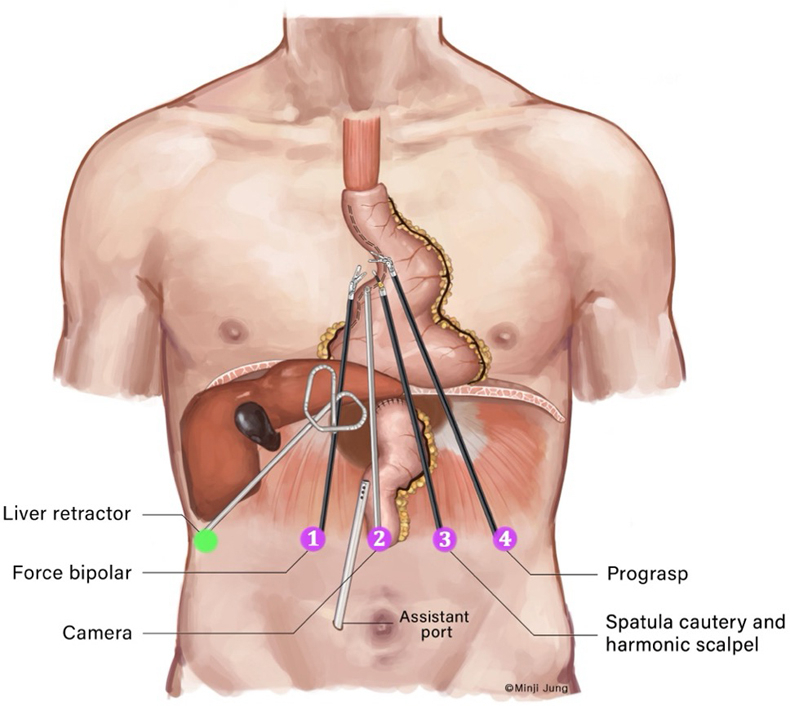
Schematic of port placement for robotic transhiatal revision of the gastric conduit.

**Central Message**

Careful patient selection and the use of the robotic platform could minimize morbidity and optimize outcomes in revisional surgery for patients with severe gastric conduit dysfunction.

Gastric conduit dysfunction after esophagectomy may markedly diminish quality of life and predispose patients to significant morbidity. In some cases, severe conduit dysfunction is linked to specific anatomic features such as dilated/redundant conduits, distal shelves, and tortuous/sigmoid-shaped conduits (Figure 1). Previously, our group reported a combined thoracoscopic and laparoscopic approach for conduit revision in 21 patients with symptomatic redundant conduits. The procedure consisted of mobilizing the conduit in the chest, reducing it within the abdomen, and securing it to the diaphragm. At a median follow-up of 12 months, 85% of patients experienced symptomatic improvement.^1^ Other institutions have also described successful symptom relief using various techniques for conduit revision.^2^, ^3^, ^4^ Herein, we present our institution's latest iteration of revisional technique: robot-assisted, totally laparoscopic, transhiatal revision of gastric conduit. This technique aims to alleviate severe postesophagectomy gastric conduit dysfunction resulting from unfavorable anatomical conditions. Informed written consent for the publication of the study was provided by the patients (institutional review board approval: STUDY20050010; initial approval date: October 5, 2020).

**Figure 1:**
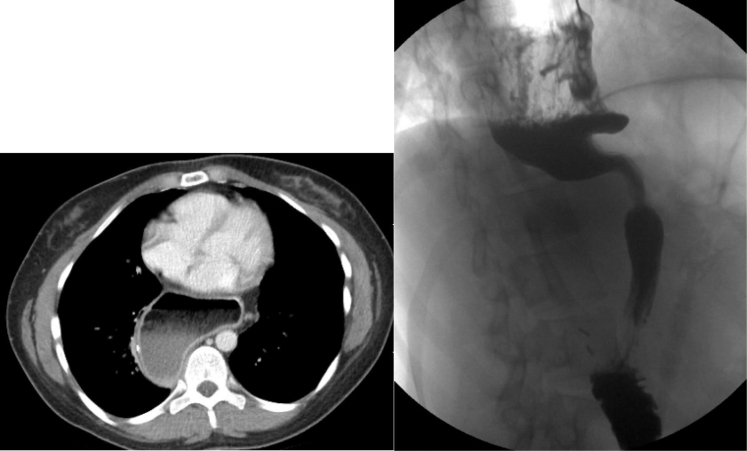
Computed tomography and barium esophagram images demonstrating a dilated and herniated gastric conduit.

## Robotic Conduit Revision: Operative Steps

Our surgical repair technique leverages the robotic platform, whose enhanced visualization and precise maneuverability in confined spaces allow the procedure to be performed entirely through laparoscopic incisions. We used Intuitive Surgical's Da Vinci Xi robotic platform for the operation, but the techniques described here may be applicable to other robotic surgery platforms. There are several key steps: intra-abdominal adhesiolysis, diaphragmatic and hiatal mobilization, high mediastinal mobilization, conduit reduction, restapling, hiatal closure, and near-circumferential conduit-pexy. The overarching goal of this revision is to reshape the dilated conduit into a narrow, straight, tubular structure that closely resembles native esophageal anatomy, thereby optimizing functional outcomes.

### Esophagogastroduodenoscopy

The procedure commences with a thorough endoscopic assessment of the conduit. This step is critical in confirming our preoperative findings before making any surgical incisions. Typically, we observe a significantly dilated conduit with sizable distal shelving overlying the thoracic gutter proximal to the diaphragmatic pinch. After this assessment, we proceed with meticulous lysis of adhesions from the previous esophagectomy.

### Port Placements

**Figure 2:**
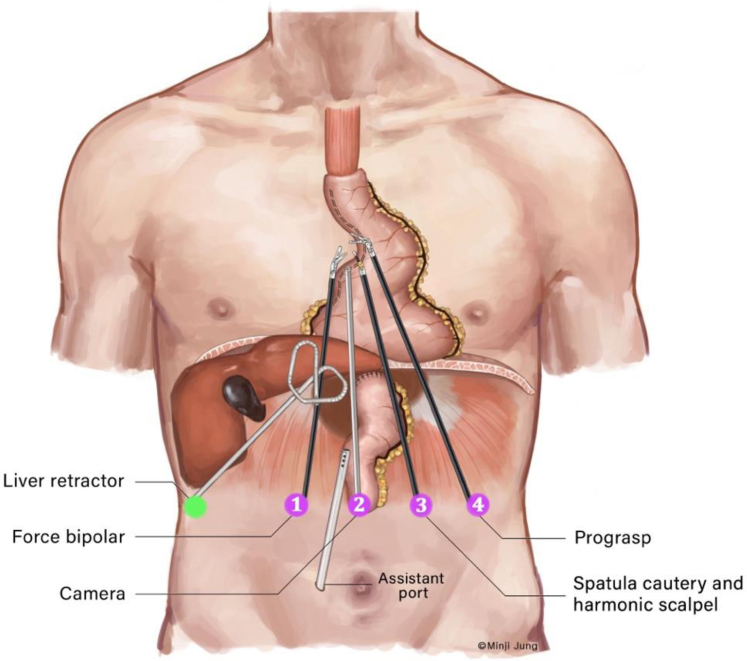
Schematic of port placements for robotic transhiatal revision of the gastric conduit after esophagectomy.

Our port-placement strategy involves a total of 6 laparoscopic ports: 4 dedicated to the robotic system, 1 accessory port, and a small port for the liver retractor (Figure 2). Our camera port is placed at the midline about 10 cm inferior to the xiphoid process (arm 2). The 2 primary working robotic arms (arms 1 and 3) are positioned equidistant from the midline at the same horizontal plane as the camera port. This strategic placement, both vertically and horizontally in proximity to the hiatus, allows the robotic arms to travel as cephalad as needed to correct the anatomical redundancy of the conduit. This positioning is crucial for complete transhiatal mobilization of the conduit, effectively obviating the need for chest incisions.

### Intra-abdominal Adhesiolysis and Hiatal Mobilization

Before docking the robot, we often use laparoscopic instruments to clear the abdominal wall at the robotic port sites. A pretzel-type Mediflex liver retractor is used to retract the liver, providing a clear view of the esophageal hiatus and crura.

Our approach to adhesiolysis follows the important principle of working from known anatomic structures to unknown areas. When mobilizing the hiatus to gain a clear view of the conduit, we begin by carefully undoing any existing sutures if there are any. We then meticulously dissect around the hiatus to mobilize the conduit and surrounding structures from the hiatal region (Video 1).

### High Mediastinal Mobilization

The next phase involves a circumferential dissection around the conduit in the posterior mediastinum. We typically initiate this dissection from the pericardial plane (anterior to the conduit) and then proceed circumferentially to the left, right, and posterior planes. When working in the mediastinum, we are mindful to minimize torque force on the crura. This is achieved by angling the robotic instruments straight through the hiatus into the mediastinum, leveraging the robotic articulations for more liberal work in the mediastinum, and using the superior visualization provided by the robotic camera (Video 2)

### Conduit Straightening/Hernia Reduction

After complete mobilization, we conduct a comprehensive assessment of the conduit. If we encounter twisting, we carefully untwist the conduit, ensuring the gastroepiploic arteries lies on the left side. In cases of redundancy or herniation, which are frequently observed, we gently reposition the conduit in the mediastinum/abdominal cavity to achieve a straight configuration from top to bottom.

### Conduit Restapling

**Figure E1:**
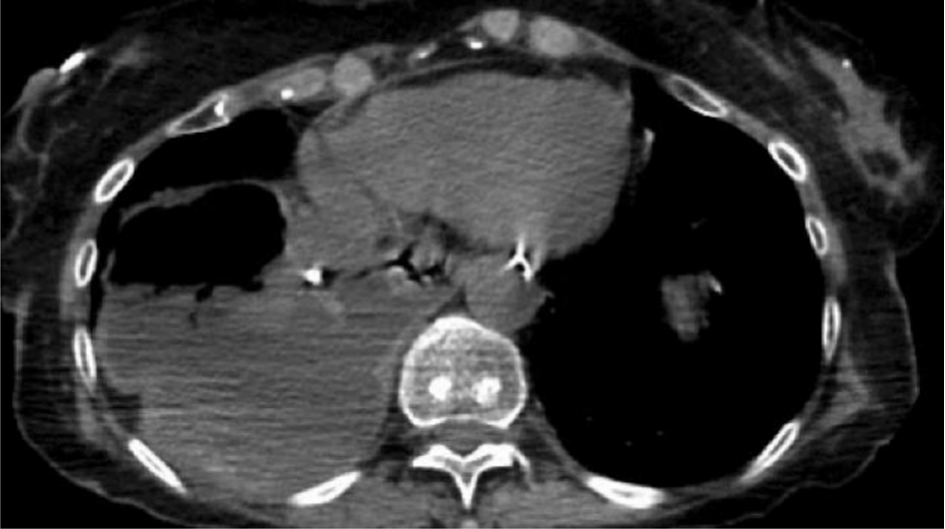
Computed tomography image showing a severely dilated and herniated gastric conduit within the right hemithorax.

The final phase involves evaluating the conduit width. If the conduit appears excessively wide and boggy, potentially inhibiting the velocity of food bolus passage and causing slow food transit and reflux, we proceed with conduit stapling (Figure E1). To prevent excessive narrowing of the lumen, we pass a 51- to 54-French (∼30 mm diameter) Maloney bougie down the esophagus. During this procedure, it is imperative to exclude strictures or luminal obstructions that may hinder safe bougie advancement. Using the robotic SureForm stapler in arm 1, we follow the curve overlying the bougie and staple the conduit from the lesser curvature, starting approximately 4 to 5 cm from the pylorus at the level of the antrum reservoir, preserving the right gastric blood supply. The stapling continues as cephalad as the anatomical redundancy of the conduit, maintaining ∼3 cm width throughout (Video 3). Robotic SureForm 45-mm black staple loads are typically used as the tissue of the conduit after esophagectomy is often thicker.

### Cruroplasty/Hiatal Closure

We then perform a cruroplasty using 0 ETHIBOND sutures (Ethicon), placing both posterior and anterior crural stitches. During this process, we use esophagogastroduodenoscopy to confirm a straight lie of the conduit without the “pinch-cock effect” of excessive narrowing at the diaphragmatic pinch. A nasogastric tube is then passed down under direct vision. Finally, we place near-circumferential 270° pexy sutures to secure the conduit to the hiatus, using a combination of 2-0 ETHIBOND and 3-0 PDS (Ethicon) sutures (Video 4). ETHIBOND sutures are used as the primary strength sutures to ensure durable cruroplasty. Additional PDS sutures are placed to reduce the risk of conduit herniation during the perioperative period, before scar tissue formation provides adequate reinforcement.

## Conclusions

The minimally invasive approach may offer reduced morbidity/mortality and holds promise as a surgical option for patients with severe gastric conduit dysfunction. Robotic revision of the gastric conduit presents a viable surgical option for patients with severe postesophagectomy conduit dysfunction. Further research is warranted to optimize conduit revision techniques.

## Conflict of Interest Statement

The authors reported no conflicts of interest.

The *Journal* policy requires editors and reviewers to disclose conflicts of interest and to decline handling or reviewing manuscripts for which they may have a conflict of interest. The editors and reviewers of this article have no conflicts of interest.

## Supplementary Data

Video 1 Hiatal mobilization. Video available at: https://www.jtcvs.org/article/S2666-2507(25)00471-7/fulltext.

Video 2 Transhiatal mediastinal dissection and conduit mobilization. Video available at: https://www.jtcvs.org/article/S2666-2507(25)00471-7/fulltext.

Video 3 Tubularizing dilated, redundant conduit by restapling along the lesser curve. Video available at: https://www.jtcvs.org/article/S2666-2507(25)00471-7/fulltext.

Video 4 Cruroplasty and near-circumferential 270° conduit pexy to the crura. Video available at: https://www.jtcvs.org/article/S2666-2507(25)00471-7/fulltext.
