# Supplementary figures and images for: Robotic transhiatal revision of gastric conduit after esophagectomy: A technical overview
Source: JTCVS Tech. 2025 Oct 30;35:102137. doi: 10.1016/j.xjtc.2025.09.038 (PMC12881808; doi:10.1016/j.xjtc.2025.09.038)

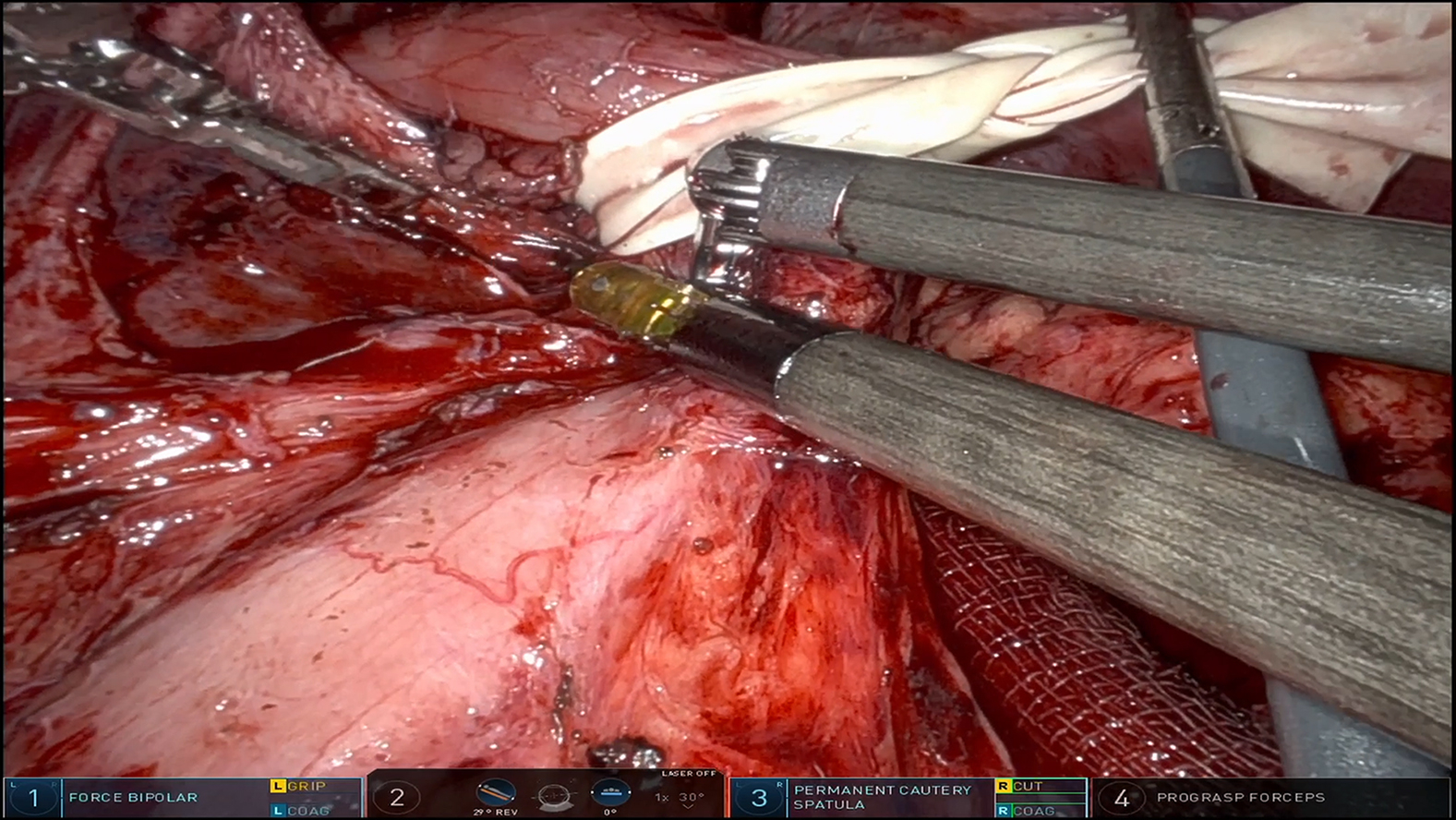

Supplement: Video 1 — Hiatal mobilization. Video available at: https://www.jtcvs.org/article/S2666-2507(25)00471-7/fulltext. [file fx2.jpg]

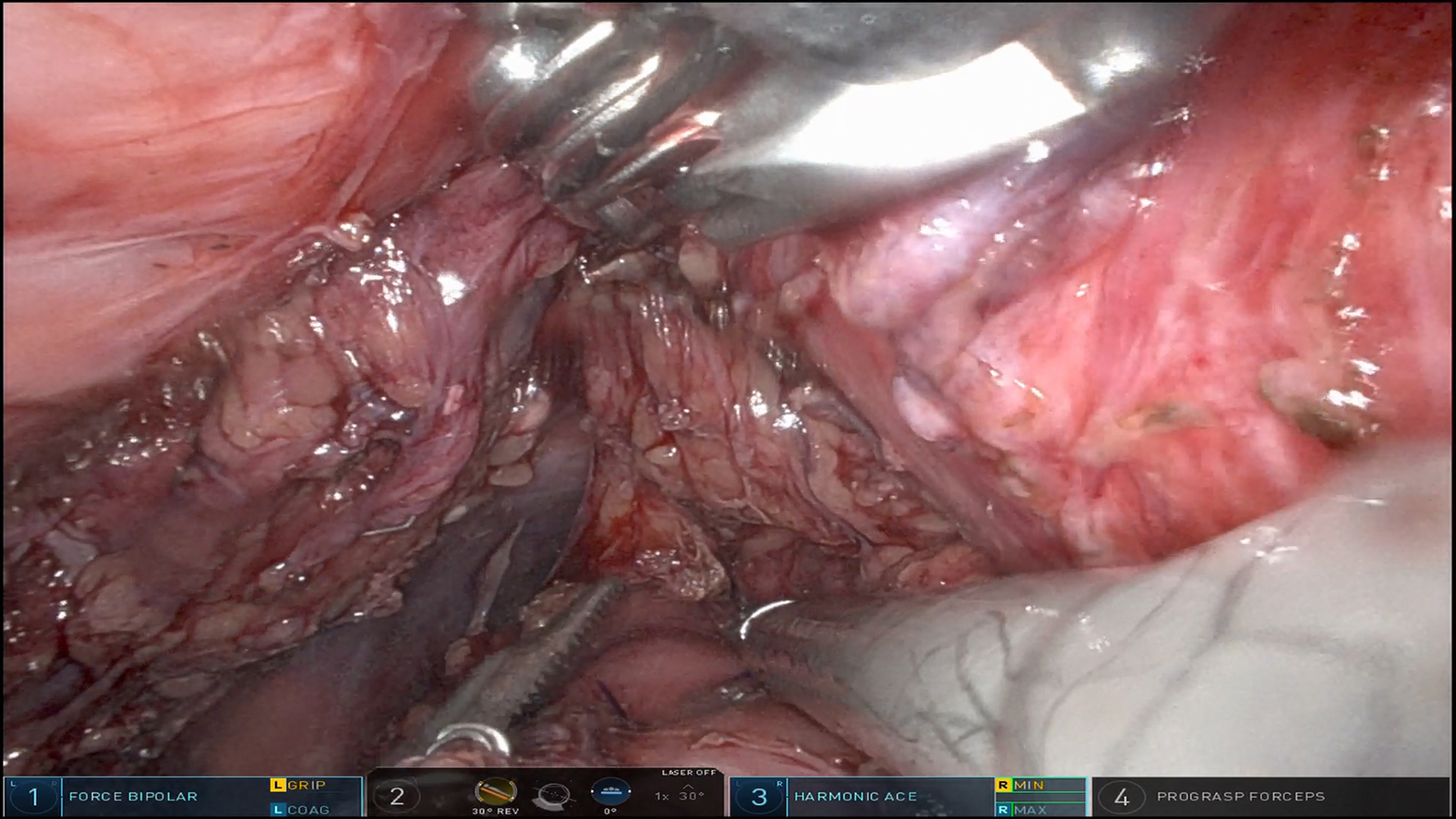

Supplement: Video 2 — Transhiatal mediastinal dissection and conduit mobilization. Video available at: https://www.jtcvs.org/article/S2666-2507(25)00471-7/fulltext. [file fx3.jpg]

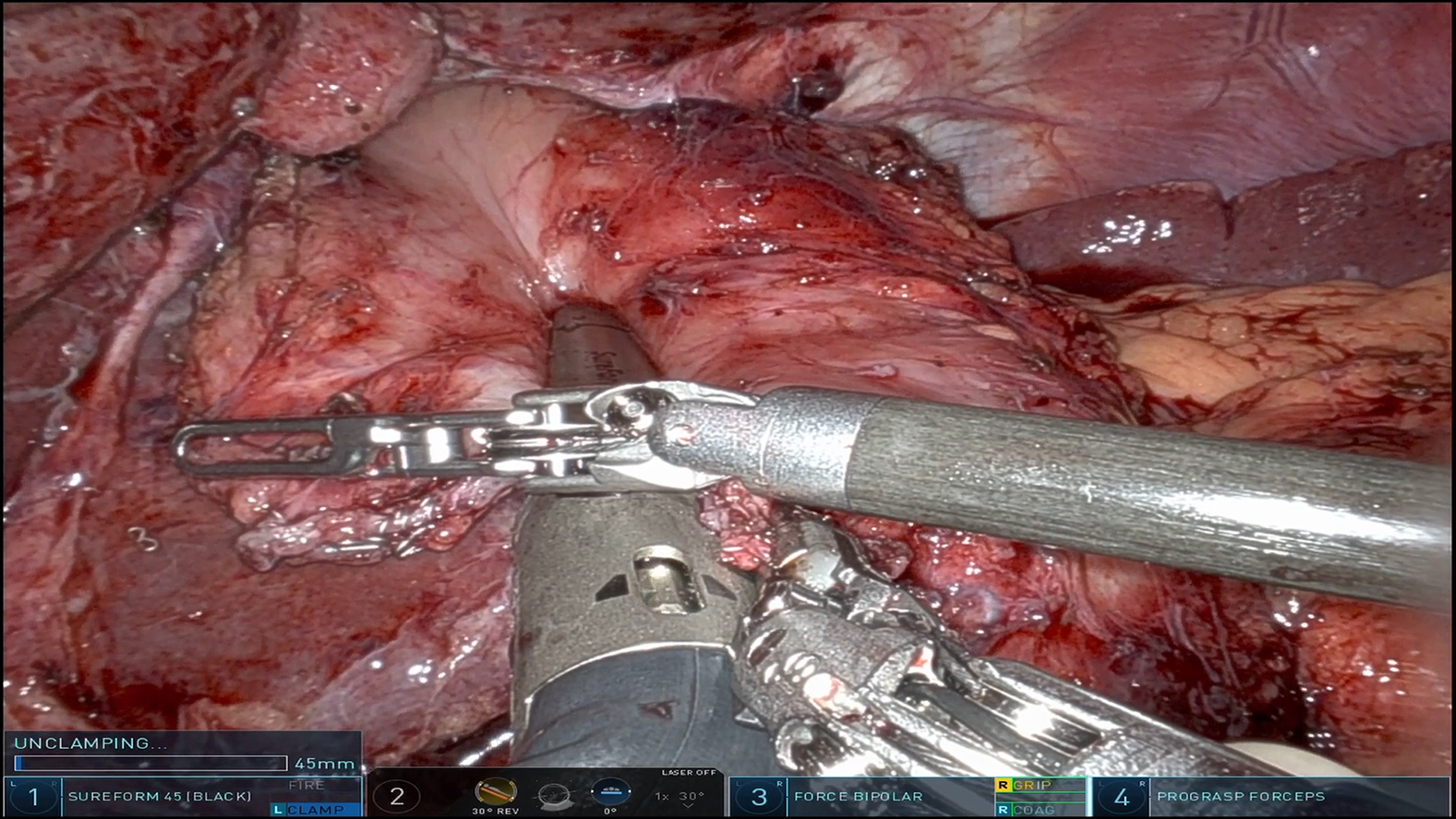

Supplement: Video 3 — Tubularizing dilated, redundant conduit by restapling along the lesser curve. Video available at: https://www.jtcvs.org/article/S2666-2507(25)00471-7/fulltext. [file fx4.jpg]

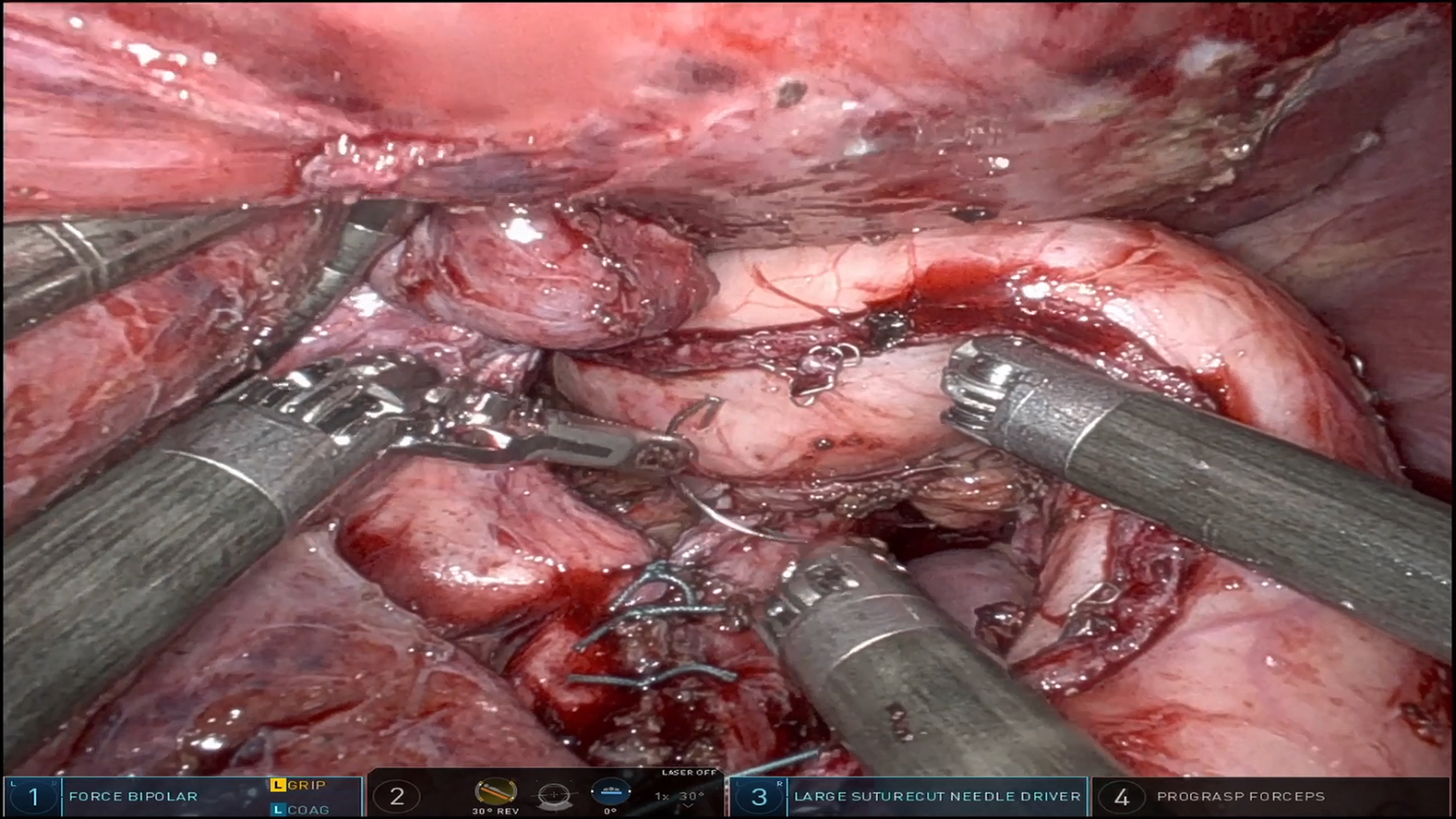

Supplement: Video 4 — Cruroplasty and near-circumferential 270° conduit pexy to the crura. Video available at: https://www.jtcvs.org/article/S2666-2507(25)00471-7/fulltext. [file fx5.jpg]
